# Wnt Signalling Promotes Actin Dynamics during Axon Remodelling through the Actin-Binding Protein Eps8

**DOI:** 10.1371/journal.pone.0134976

**Published:** 2015-08-07

**Authors:** Eleanna Stamatakou, Monica Hoyos-Flight, Patricia C. Salinas

**Affiliations:** Department of Cell and Developmental Biology, University College London, London, WC1E 6BT, United Kingdom; University of Texas Medical Branch, UNITED STATES

## Abstract

Upon arrival at their synaptic targets, axons slow down their growth and extensively remodel before the assembly of presynaptic boutons. Wnt proteins are target-derived secreted factors that promote axonal remodelling and synaptic assembly. In the developing spinal cord, Wnts secreted by motor neurons promote axonal remodelling of NT-3 responsive dorsal root ganglia neurons. Axon remodelling induced by Wnts is characterised by growth cone pausing and enlargement, processes that depend on the re-organisation of microtubules. However, the contribution of the actin cytoskeleton has remained unexplored. Here, we demonstrate that Wnt3a regulates the actin cytoskeleton by rapidly inducing F-actin accumulation in growth cones from rodent DRG neurons through the scaffold protein Dishevelled-1 (Dvl1) and the serine-threonine kinase Gsk3β. Importantly, these changes in actin cytoskeleton occurs before enlargement of the growth cones is evident. Time-lapse imaging shows that Wnt3a increases lamellar protrusion and filopodia velocity. In addition, pharmacological inhibition of actin assembly demonstrates that Wnt3a increases actin dynamics. Through a yeast-two hybrid screen, we identified the actin-binding protein Eps8 as a direct interactor of Dvl1, a scaffold protein crucial for the Wnt signalling pathway. Gain of function of Eps8 mimics Wnt-mediated axon remodelling, whereas Eps8 silencing blocks the axon remodelling activity of Wnt3a. Importantly, blockade of the Dvl1-Eps8 interaction completely abolishes Wnt3a-mediated axonal remodelling. These findings demonstrate a novel role for Wnt-Dvl1 signalling through Eps8 in the regulation of axonal remodeling.

## Introduction

The arrival of axons at their synaptic targets results in extensive remodelling of the growth cone and the terminal portions of the axon leading to the formation of terminal branches and presynaptic boutons. This extensive remodelling is crucial for the formation of synaptic boutons and requires coordinated changes in the organisation and dynamics of both the actin and microtubule cytoskeletons [1–5]. Target-derived cues promote terminal remodelling of axons, but little is known about how these extracellular signals influence the cytoskeleton.

Wnt secreted molecules play important roles in the formation of neuronal circuits by regulating axon pathfinding and remodelling, dendritic development and synapse assembly [6–11]. Wnts act as target-derived signalling molecules that promote axon terminal remodelling and the subsequent assembly of presynaptic boutons [12–14]. In the cerebellum, Wnt7a released by granule cell neurons acts on incoming mossy fiber axons to induce growth cone enlargement and axonal spreading, processes that are accompanied by the recruitment of presynaptic components [12]. Importantly, Wnt7a deficient mice exhibit defects in axonal terminal remodelling and the accumulation of synaptic proteins at mossy fibre axons [12,15]. In the fly neuromuscular junction (NMJ), Wg, another member of the Wnt family, is required for the proper formation of synaptic boutons [16]. In the spinal cord, motorneuron-derived Wnt3 promotes the axonal terminal remodelling of NT-3 responsive dorsal root ganglia (DRG) neurons [13]. Therefore, Wnt proteins are target-derived signals that induce extensive structural remodelling of presynaptic axonal terminals.

During axon remodelling, Wnts induce profound changes in the organisation of microtubules (MTs). In the presence of Wnts, MTs extend towards the leading edge of the growth cone, but their direction is severely affected resulting in the formation of looped MTs [12,14]. Wnt3a acts through a divergent canonical β-catenin pathway that is independent of transcription, but requires Dishevelled-1 (Dvl1) and Glycogen synthase kinase 3β (Gsk3β) inhibition to regulate MT looping [14]. This pathway directly signals to the cytoskeleton by inducing loss of APC from the MT plus-ends, resulting in defects in the directionality of MT growth [14]. Importantly, studies at the *Drosophila* NMJ revealed that the divergent canonical Wnt pathway through Shaggy/Gsk3 promotes axonal remodelling manifested by the formation of satellite boutons and the presence of looped microtubules [17,18]. Consistently, *wg* mutants have defects in synaptic bouton formation and morphology [16,18]. Together, these studies demonstrate that Wnt signalling factors target the microtubule cytoskeleton to drive axons to their synaptic targets. However, the effects of Wnts on axonal morphology, such as axonal spreading and growth cone enlargement, suggest that Wnts might also modulate the actin cytoskeleton.

The actin cytoskeleton is regulated by a number of actin-binding proteins (ABPs) that control nucleation, severing, cross-linking, and capping of actin filaments, as well as monomer sequestering. Although a large number of ABPs are present at growth cones, only few have been examined in axon guidance and target recognition [2,4,19,20]. Eps8 (epidermal growth factor receptor pathway substrate 8) is a multi-functional actin-binding protein that regulates the actin cytoskeleton through diverse mechanisms [21–24]. Eps8 binds to filamentous actin and directly modulates actin dynamics through its barbed-end capping and bundling activities [22,25]. Eps8 can also regulate the actin cytoskeleton indirectly via tyrosine receptor-mediated Rac1 activation [24,26,27]. Therefore, Eps8 modulates both actin dynamics and organisation. In neurons, Eps8 is prominently enriched in axonal growth cones and dendritic spines where it regulates filopodium and spine formation through its capping activity [28–30]. However, its role on axonal terminal remodelling remains elusive.

Here, we examined the mechanism by which Wnt signalling regulates the actin cytoskeleton during terminal axon remodelling. We show that Wnt3a causes a rapid accumulation of F-actin in growth cones. Time-lapse recordings of neurons expressing GFP-actin revealed that Wnt3a promotes lamellar protrusion and enhances filopodia movement speed in growth cones, processes that are both mediated by increased actin dynamics. In addition, we show that expression of Dvl1 or Gsk3β inhibition mimic the effect of Wnt3a in F-actin accumulation. Using a yeast-two hybrid screen, we identified Eps8, a multifunctional actin-regulating protein, as a direct interactor of Dvl1. Gain of function of Eps8 mimics Wnt3a-mediated growth cone remodelling, whereas loss of function of Eps8 impairs Wnt3a-induced axonal remodelling. Importantly, we show that the interaction of Dvl1 and Eps8 is required for Wnt3a-mediated axon remodelling. Our studies identify Eps8 as a novel target for Wnt signalling during axonal remodeling.

## Results

### Wnt3a promotes accumulation of F-actin in growth cones

**Fig 1:**
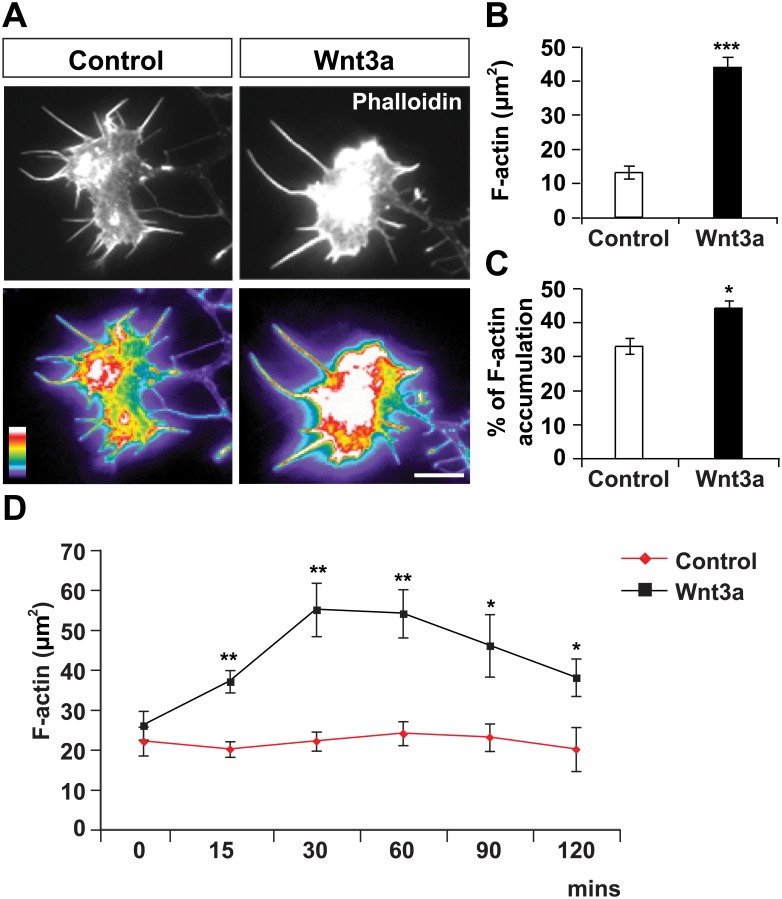
Wnt3a increases F-actin levels in growth cones. (A) Two hours treatment with Wnt3a induces F-actin accumulation in growth cones of DRG neurons. Pseudocolor images of growth cones labeled with Phalloidin (bottom panels) highlight the differences in F-actin levels between Wnt3a and control-treated cells. Two growth cones of similar size are shown for illustrative purposes. Scale bar: 10 μm. (B) The area of growth cones with bright F-actin staining (red and white in pseudocolor images) is larger in Wnt3a-treated growth cones than in controls. (C) Growth cone area with bright F-actin was normalized to total growth cone area. (D) Time-course experiments demonstrate that Wnt3a significantly increases F-actin content in growth cones within 15 mins. *p<0.05, **p<0.01, ***p<0.001.

Our previous studies showed that Wnt3a reduces axonal outgrowth within 20 minutes, but induces changes in microtubule organisation and growth cone enlargement by 60 minutes [14]. Thus, Wnt3a affects growth cone behaviour before changes in microtubule organization are evident. We therefore examine the possible contribution of the actin cytoskeleton to Wnt-mediated axonal remodelling. Here, we investigated the impact of Wnt3a in DRG neurons on the accumulation and distribution of filamentous actin (F-actin) (Fig 1A). We found that Wnt3a significantly increased F-actin levels in growth cones, as determined by the increase in the area of highly-intense Phalloidin staining (red and white in pseudocolor images; Fig 1A and 1B). Given the significant effect of Wnt3a on growth cone size, we normalised the area of F-actin accumulation to growth cone area (% F-actin accumulation) and found that indeed Wnt3a increases the percentage of F-actin content in growth cones (Fig 1C). We next determined the time required for Wnt3a to promote F-actin accumulation in growth cones. Time-course experiments of DRG neurons treated with Wnt3a revealed that the F-actin accumulation in growth cones occurred within 15 minutes (Fig 1D). These findings demonstrate that Wnt3a rapidly increases the content of F-actin in growth cones before inducing any changes in growth cone size and microtubule reorganisation [14].

### Wnt3a increases actin dynamics during axon remodelling

**Fig 2:**
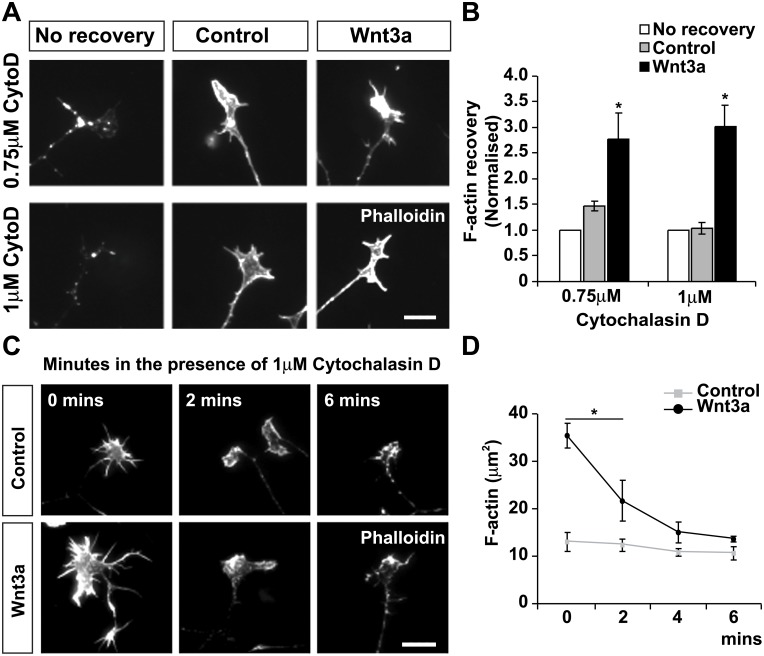
Wnt3a increases actin dynamics in growth cones. (A) DRG neurons were treated different concentrations of with CytoD and then recovered in control or Wnt3a growth media. Scale bar: 10μm. (B) Quantification shows that F-actin levels are after recovery are significantly higher in the presence of Wnt3a. Values indicate F-actin levels relative to growth cones fixed immediately after treatment with CytoD (no recovery). (C) Wnt3a treatment induces accumulation of F-actin in growth cones, which are rapidly dissembled by after short exposure to 1mM CytoD. Scale bar: 10μm. (D) F-actin content in growth cones does not significantly decrease in control conditions in the presence of CytoD over the period of 6 mins. In contrast, F-actin content is significantly decreased in Wnt3a-treated growth cones within 2 mins. *p<0.05, **p<0.01, ns: non-significant.

The effect of Wnt3a on F-actin content could be due to an increase in the rate actin polymerisation or decrease in actin depolymerisation. To determine which of these processes are regulated by Wnt3a, we first performed F-actin recovery experiments [31] using Cytochalasin-D (CytoD), a pharmacological inhibitor of actin subunit assembly at the filament barbed ends [32,33]. DRG neurons were treated with two different concentrations of CytoD for 20 minutes to induce actin disassembly, followed by a 30 minutes recovery period in the presence of control medium or Wnt3a, during which F-actin would be re-assembled (Fig 2A). When neurons treated with 0.75 μM CytoD were allowed to recover in control media, a 1.5-fold increase in the intensity of F-actin was observed in growth cones (Fig 2A). However, when neurons recovered in the presence of Wnt3a a 3-fold increase in the intensity of F-actin was observed in growth cones (Fig 2A and 2B). Consistently, neurons treated with 1 μM CytoD showed very little recovery in F-actin intensity when recovered in control growth medium, yet a significant recovery was observed in the presence of Wnt3a (Fig 2A and 2B). These results demonstrate that Wnt3a promotes actin filament assembly or polymerization in growth cones.

To determine whether Wnt3a also affects actin dynamics, neurons were first exposed to control medium or Wnt3a for 2 hours followed by a short exposure to 1 μM CytoD for 2, 4 and 6 mins (Fig 2C). CytoD did not significantly change the area of highly-intense F-actin content in control-treated growth cones within the first 6 minutes (Fig 2D). However, filopodia started to disappear and F-actin aggregates begun to form (Fig 2C), as previously reported [34], demonstrating that CytoD was effective. Interestingly, in Wnt3a-treated growth cones CytoD induced a significant decrease in the area of highly-intense F-actin content within the first 2 minutes of application (Fig 2C and 2D), resulting in a progressive and rapid decline in F-actin content back to control levels. These results indicate that Wnt3a promotes the assembly of highly dynamic actin filaments that are quickly dissembled in the presence of CytoD.

**Fig 3:**
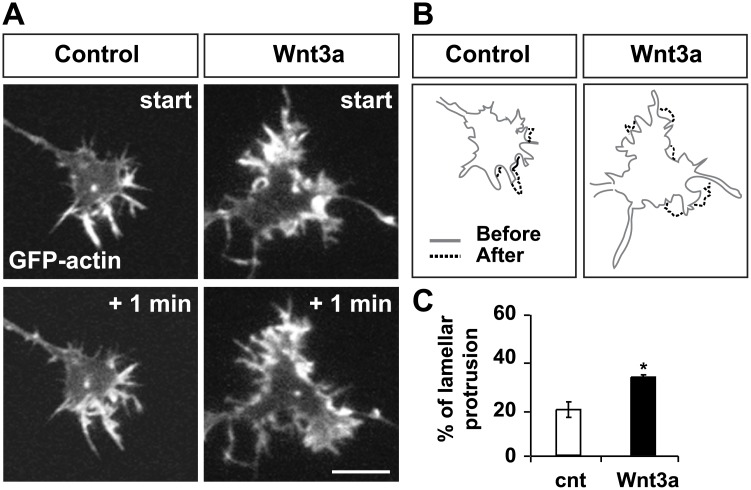
Wnt3a increases lamellar protrusion. (A) Still frames captured 1 min apart from time-lapse recordings of GFP-β-actin expressing neurons exposed to control or Wnt3a medium for 16 hours. Scale bar: 5μm. (B) Tracings of lamella undergoing protrusion (protruded perimeter depicted in dashed line). (C) Quantification shows that Wnt3a significantly increases the percentage of lamella undergoing protrusion. *p<0.05.

To gain further insight into the effect of Wnt3a on the actin cytoskeleton, we performed time-lapse recordings of DRG neurons expressing GFP-β-actin to visualise of the actin cytoskeleton in real time. Lamellar protrusion and filopodia velocity were examined in neurons exposed to control medium or Wnt3a (Fig 3A, S1 Movie and S2 Movie). We observed that Wnt3a significantly increased the percentage of lamellar perimeter undergoing protrusion in growth cones compared to controls (Fig 3A–3C, S1 Movie and S2 Movie). In addition, the speed of filopodia movement was significantly higher in neurons exposed to Wnt3a compared to controls (control: 0.022 ± 0.0036 **μ**m/sec; Wnt3a: 0.035 ± 0.002 **μ**m/sec, p<0.05). These results further suggest that Wnt3a promotes actin polymerisation.

### Wnt3a modulates axonal actin through Dvl1 and Gsk3β

**Fig 4:**
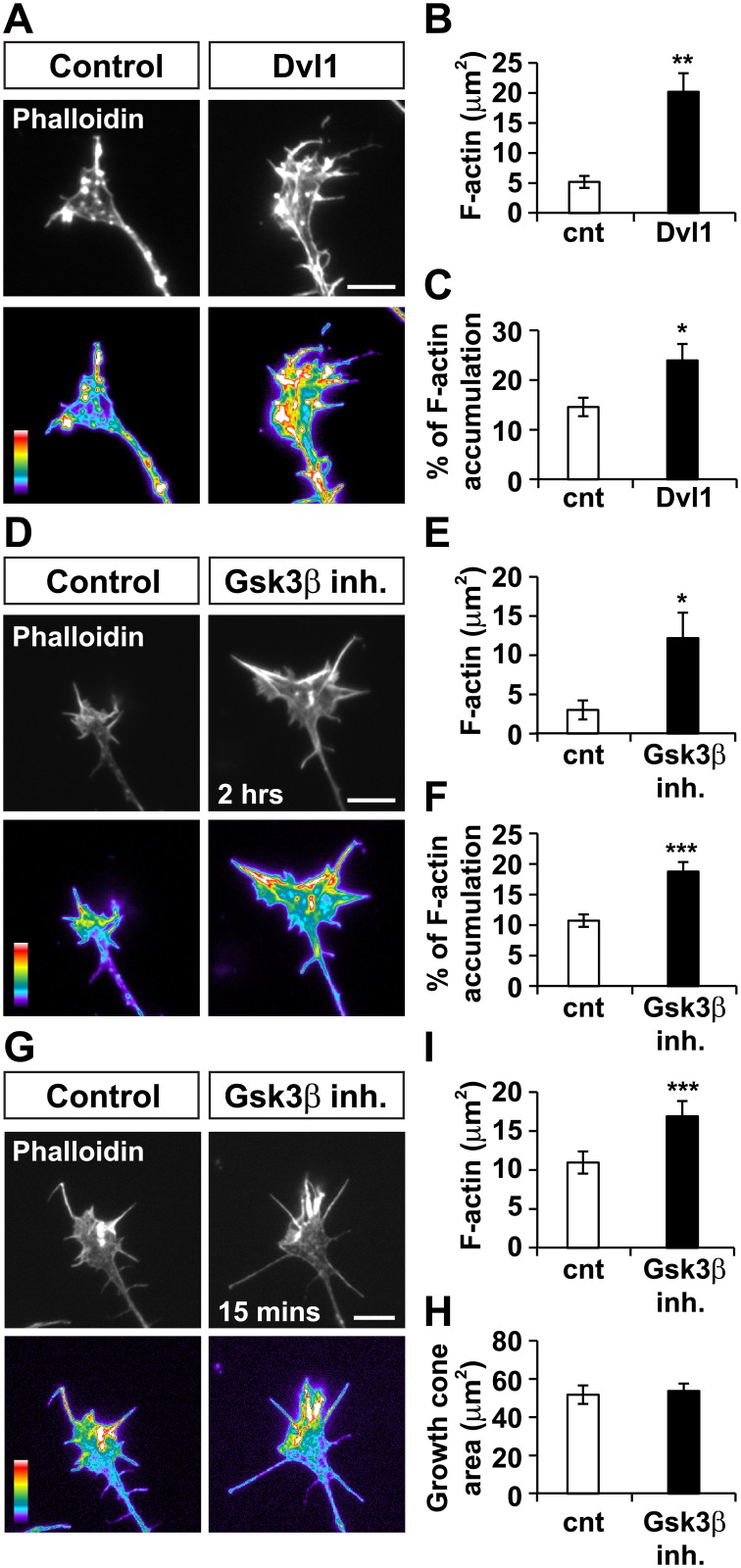
Dvl1 gain of function and inhibition of Gsk3β mimic the effect of Wnt3a in F-actin accumulation. (A) Dvl1 gain of function induces F-actin accumulation in growth cones. Pseudocolor images of growth cones labeled with phalloidin show significant differences in F-actin levels between Dvl1-expressing and control GFP-expressing cells. Scale bar: 5μm. (B and C) Dvl1 increases the area of growth cones with bright F-actin staining and also induces the percentage of growth cone area containing high levels of F-actin. (D) A specific Gsk3β peptide inhibitor promotes accumulation of F-actin in growth cones. Scale bar: 5μm. (E) Inhibition of Gsk3β induces F-actin accumulation and (F) the percentage of growth cone area with bright F-actin fluorescence. (G-H) Acute expose to the Gsk3β peptide inhibitor promotes accumulation of F-actin in growth cones, without affecting growth cone size. Scale bar: 5μm. *p<0.05, **p<0.01, ***p<0.001.

To determine how Wnt3a regulates actin dynamics, we examined the role of Dvl1 and Gsk3β as these two Wnt signalling components contribute to Wnt-mediated axonal remodelling [14]. Our previous studies showed that Wnt3a failed to induce axon remodelling in Dvl1 mutant DRG neurons [14]. We therefore performed gain of function experiments to examine in detail the effect of Dvl1 on actin. As we observed with Wnt3a, gain of function of Dvl1 significantly increased F-actin accumulation in growth cones (Fig 4A, 4B and 4C).

Given the crucial role of Gsk3 in axon remodelling [14], we next examined whether Gsk3β inhibition promotes accumulation of F-actin like Wnt3a (Fig 4D). We found that a cell-permeable peptide inhibitor, that specifically blocks Gsk3β [35], significantly increased F-actin accumulation in growth cones (Fig 4E and 4F) when applied for 2 hrs. To determine whether Gsk3**β** inhibition acts on the same time-frame with Wnt3a and whether F-actin accumulation precedes Gsk3**β**-mediated growth cone remodelling, we treated DRG neurons with the cell-permeable Gsk3**β** peptide inhibitor for 15 mins (Fig 4G). We found that acute inhibition of Gsk3**β** did significantly induce F-actin accumulation in growth cones without affecting growth cone size (Fig 4I and 4H). Together, these results suggest that Wnt3a regulates actin dynamics through Dvl1 and inhibition of Gsk3β.

### Dvl1 interacts with the actin-binding protein Eps8

**Fig 5:**
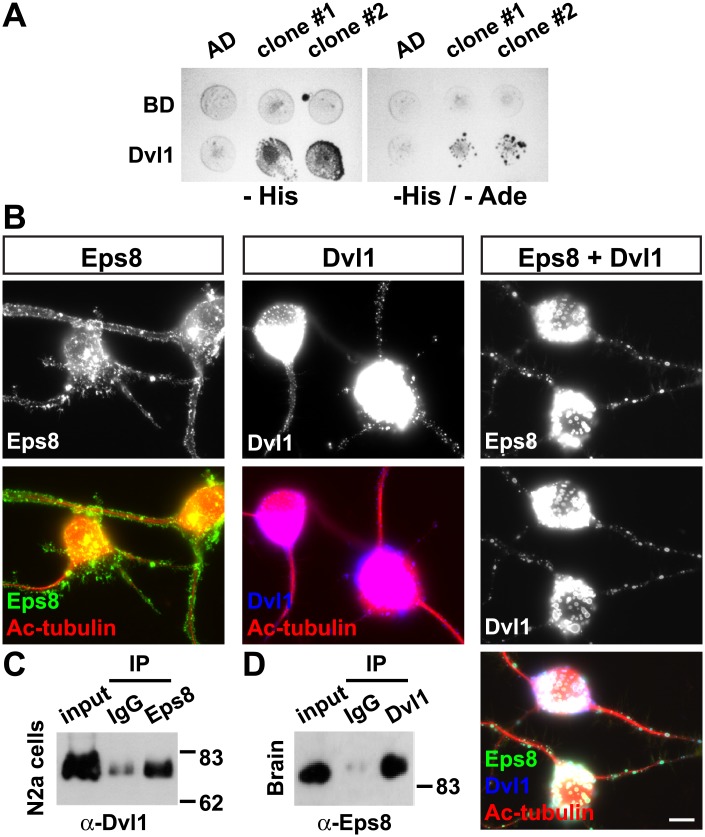
Dvl1 interacts with the actin-binding protein Eps8. (A) Dvl1 was fused to the GAL4 DNA binding domain (BD) and tested for interactions against an empty vector or Eps8L3 fused to the GAL4 activation domain (AD). Growth on-His and-His/-Ade media shows a strong and direct interaction of Dvl1 with Eps8L3, but not with the empty vector (AD). (B) The presence of Dvl1 changes the cellular localisation of Eps8 into defined puncta also containing Dvl1 in N2a cells. Scale bar: 10μm. (C) Co-immunoprecipitation experiments show the interaction between Dvl1 and Eps8 in N2a cells. (D) Immunoprecipitation experiments using brain lysates from P24 mice show that endogenous Eps8 interacts with endogenous Dvl1.

To further unravel the mechanisms by which Wnt3a regulates the actin cytoskeleton during axonal remodelling, we performed a yeast two-hybrid screen to identify molecules that interact with Dvl1 and regulate the actin cytoskeleton. We found that Eps8L3, a member of the Eps8 family of actin-binding proteins, showed a direct interaction with Dvl1 (Fig 5A). Importantly, this interaction was strong, as revealed by the stringent nutrition selection (lacking Histidine and Adenine) (Fig 5A). Given the lack of commercial antibodies for Eps8L3, we focused our studies on Eps8, another member of this family of actin-capping proteins, which is most highly expressed in the nervous system [23] and has been previously shown to bind Dvl1 [36].

Previous studies showed that Eps8 recruits XDsh (the Xenopus homologue of Dvl1) to the plasma membrane and actin filaments in animal cap cells [37]. To examine whether Eps8 can affect the localization of Dvl1 in neuronal cells, we co-expressed Eps8 and Dvl1 in differentiated Neuroblastoma 2a (N2a) cells. When expressed alone, Eps8 had a membrane and punctate localisation (Fig 5B). In contrast, in the presence of Dvl1, Eps8 co-localised with Dvl1 puncta and its localization to the membrane decreased (Fig 5B). These results suggest that Eps8 might be recruited to Dvl1 sites. Moreover, co-immunoprecipitation experiments demonstrated that these proteins interacted in N2a neuronal cells (Fig 5C). Importantly, in the adult mouse brain, we found an interaction between endogenous Dvl1 and Eps8 using immunoprecipitation (Fig 5D). Collectively, these results demonstrate that Dvl1 interacts with Eps8 in neurons and that Dvl1 may regulate the distribution and/or function of Eps8.

### Eps8 is required for Wnt3a-mediated axonal remodelling

**Fig 6:**
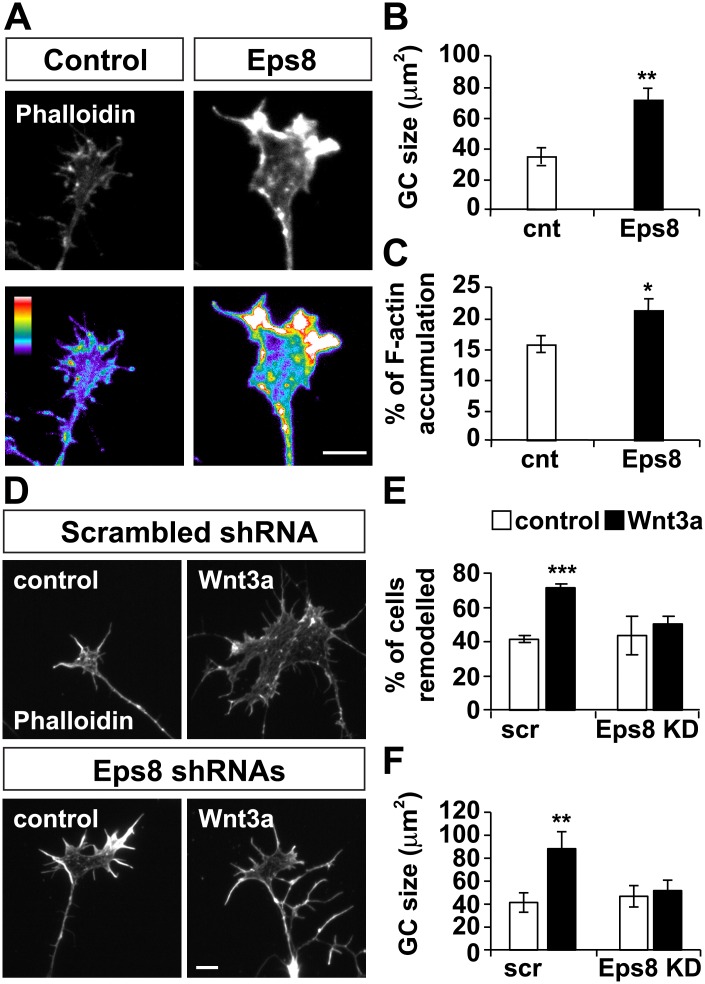
Eps8 is required for Wnt3a-mediated axonal remodeling. (A) Eps8 promotes growth cone enlargement and increases F-actin accumulation in growth cones. Pseudocolor images show the different levels of F-actin in growth cones of Eps8-expressing and control GFP-expressing cells. Scale bar: 5μm. (B and C) Growth cone area is larger in Eps8-expressing cells than in controls cells, as is the percentage of growth cone area with high F-actin fluorescence. (D) DRG neurons expressing scramble or Eps8 shRNAs were treated with Wnt3a for 2 hrs. Scale bar: 5μm. (E) In scrambled shRNA-expressing cells Wnt3a increases the percentage of cells that show axonal remodelling (% of cells remodelled) and (F) promotes growth cone enlargement. In contrast, neurons expressing Eps8shRNAs do not respond to Wnt3a. *p<0.05, **p<0.01, ***p<0.001.

As Eps8 is an actin-binding protein and interacts with Dvl1, this protein could mediate the effect of Wnt-mediated axon remodelling, in particular the changes in actin cytoskeleton. To examine this possibility, we first performed gain of function studies and found that Eps8 induced axonal remodelling manifested by a significant increase in growth cone size when compared to control GFP-expressing cells (Fig 6A and 6B). We also found that like with Wnt3a or gain of function of Dvl1, Eps8 induced a significant increase in F-actin accumulation (Fig 6A and 6C). Next, we examined whether Eps8 induces changes in microtubule organisation, as we have previously shown for the Wnt3a-Dvl1-Gsk3**β** pathway [14]. In control GFP-expressing DRG neurons, microtubules had a splay morphology (S2 Fig). However, Eps8-expressing DRG neurons had enlarged growth cones with looped microtubules (S2 Fig), as observed with exposure to Wnt3a, expression of Dvl1 or inhibition of Gsk3**β** [14]. Thus, Eps8 mimics the effect of Wnt3a and Dvl1 gain of function on axonal remodelling by affecting both the actin and microtubule cytoskeleton.

Next we performed loss of function studies using shRNA-mediated Eps8 knockdown (Eps8 KD) (Fig 6D). To obtain a significant level of knockdown, DRG neurons were transfected with a combination of three different shRNAs that specifically target Eps8 [29]. Neurons expressing scrambled control shRNA construct were used as controls. To first verify that we efficiently silenced Eps8, we quantified the level of endogenous Eps8 in growth cones and found that expression of Eps8 shRNAs promoted a significant reduction in Eps8 protein levels (S3 Fig). In addition, we analysed the number of axonal filopodia, as previous studies have shown that loss of Eps8 results in increased filopodium density in both axons and dendrites [28,29]. Indeed, we observed that cells expressing shRNAs against Eps8 exhibited a 48% increase in axonal filopodium density (S3 Fig), demonstrating a significant loss of function effect of Eps8 [28,29]. We then examined the impact of Eps8 loss of function on Wnt3a-induced growth cone remodelling. We found that Wnt3a induced axonal remodelling in control “scrambled” shRNA-expressing cells by increasing both the percentage of cells that showed enlarged growth cones (% of cells remodelled; Fig 6E) and the average growth cone area (Fig 6F), as we have previously shown [14]. In contrast, Eps8 shRNA-expressing neurons did not remodel in the presence of Wnt3a (Fig 6D–6F). These experiments demonstrate that Eps8 is required for Wnt3a signalling to induce axonal remodelling.

To determine the role of the interaction of Dvl1 and Eps8 in Wnt3a-mediated axonal remodelling, we decided to interfere with the binding between these two proteins. A previous study showed that Eps8 interacts with the PDZ domain of Dvl1 [36], a conserved region important for protein-protein interactions and for Wnt signalling [38–42]. We therefore decided to examine the contribution of the PDZ domain of Dvl1 to Eps8 function.

**Fig 7:**
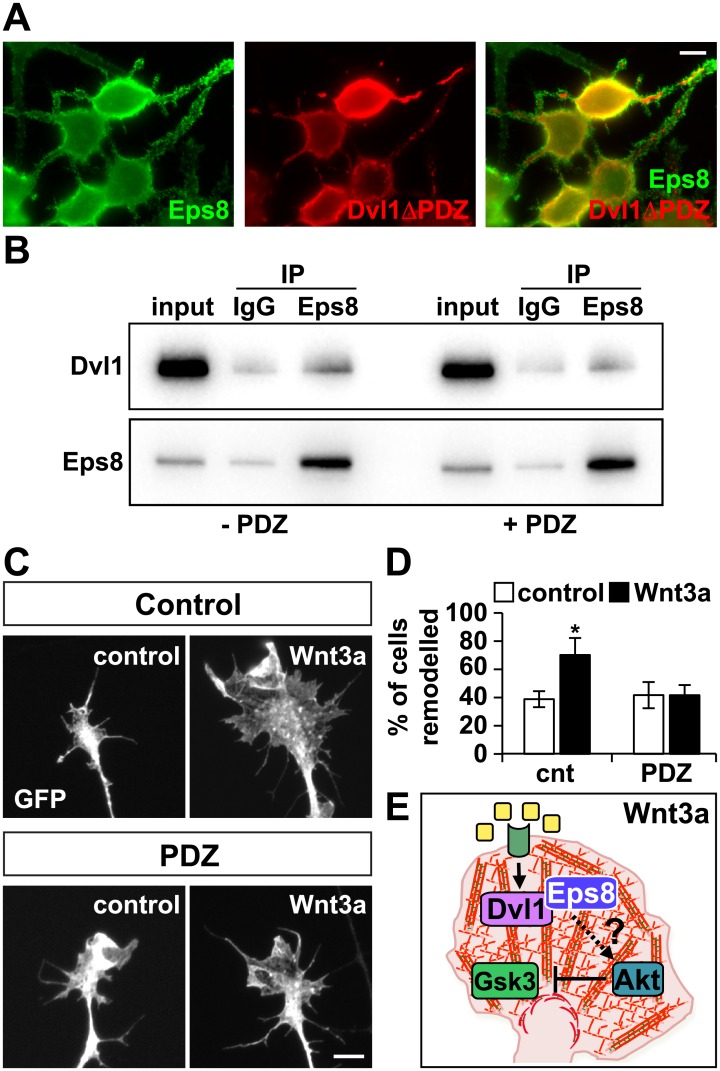
The interaction between Dvl1 and Eps8 is required for Wnt3a-mediated axonal remodeling. (A) Dvl1ΔPDZ does not affect the localisation of Eps8 in N2a cells. (B) Expression of the PDZ domain of Dvl1 in N2a cells decreases the binding of Dvl1 and Eps8 by 38%, as assessed by co-immunoprecipitation experiments. (C) DRG neurons expressing the PDZ domain of Dvl1 were treated with Wnt3a for 2 hrs. Scale bar: 5μm. (D) In GFP-expressing cells Wnt3a increases the percentage of cells that show axonal remodelling (% of cells remodelled). In contrast, neurons expressing the PDZ domain of Dvl1 do not remodel in the presence of Wnt3a. (E) Proposed model for the role of Eps8 in Wnt3a-mediated axonal remodelling. Wnt3/3a induces the formation of highly dynamic actin filaments and growth cone enlargement. These effects are mediated though the direct interaction of Dvl1 and Eps8, and Gsk3β inhibition possibly induced by Eps8-Akt signalling. *p<0.05.

First, we co-expressed Eps8 and a mutant Dvl1 that lacks the PDZ domain (Dvl1ΔPDZ) in differentiated N2a cells. We found that Dvl1ΔPDZ was not able to change the localisation of Eps8 (Fig 7A), in sharp contrast to full length Dvl1 (Fig 5B). These results suggest that the redistribution of Eps8 by Dvl1 depends on the PDZ domain of Dvl1. Next, we reasoned that the PDZ domain of Dvl1 could block Dvl1-Eps8 interaction and therefore interfere with its function in Wnt-mediated remodelling. To examine this, we co-expressed full length Eps8, Dvl1 with the PDZ domain of Dvl1 in N2a cells and performed co-immunoprecipitation experiments. We found that the PDZ domain substantially decreased the interaction between Eps8 and Dvl1 (Fig 7B). Thus, the PDZ domain of Dvl1 could be used as a tool to examine the role of Dvl1-Eps8 interaction in axonal remodelling.

Next, we investigated the contribution of the interaction of Dvl1 with Eps8 on Wnt-mediated axon remodelling. We found that DRG neurons expressing the PDZ domain of Dvl1 were unable to respond to Wnt3a, in contrast to control neurons that showed enlarged growth cones upon exposure to Wnt3a (% of cells remodelled; Fig 7C and 7D). These findings demonstrate that the interaction between Dvl1 and Eps8 is required for growth cone remodelling induced by Wnt3a.

## Discussion

Here we demonstrate that Wnt signalling promotes axon remodelling, a process that precedes the formation of synaptic boutons, by controlling the dynamics of the actin cytoskeleton in addition to its previous described role in microtubule re-organisation. Our previous studies showed that Wnt3a induces axonal remodelling through changes in the organisation and dynamics of microtubules [13,14]. In this study, we now show that Wnt3a also promotes profound changes in the actin cytoskeleton during axon remodelling through a pathway that involves the scaffold protein Dvl1, Eps8, an actin-binding protein that directly interacts with Dvl1, and inhibition of Gsk3**β**. Importantly, Eps8 and its interaction with Dvl1 is required for Wnt3a-mediated axonal remodelling. These findings reveal a novel role for Eps8 downstream of Wnt signalling to regulate cytoskeleton remodelling.

During axon remodelling, Wnt3a induces growth cone pausing within the first 20 minutes accompanied by an increase in membrane protrusion in growth cones and at the distal portion of the axon [14]. This effect is followed by the loss of APC from microtubule plus ends at 30 mins, growth cone enlargement and the formation of looped microtubules. Although, growth cones start to increase in size after 30 mins, the full effect of Wnts on growth cone enlargement is evident after 60 mins [14]. Here we showed that Wnt3a also promoted accumulation of F-actin in growth cones within 15 minutes and therefore before the time when MTs or growth cone size are affected. Interestingly, we have previously shown that blocking actin dynamics with low levels of Latrunculin B does not fully impair Wnt3a-mediated growth cone remodelling [14]. This result originally indicated that actin dynamics is not critical for Wnt3a-mediated remodelling. However, our new findings clearly demonstrated that Wnt3a induced profound changes in the actin cytoskeleton prior to growth cone remodelling, suggesting that actin dynamics is also important for Wnt3a-mediated axonal remodelling. These apparent contradictory results could be explained by the fact that Latrunculin B binds to and sequesters actin monomers in a molar ratio of 1:1 [43]. If used at low concentration, Latrunculin B will only affect a small fraction of the actin monomer pool present at growth cones. Thus, Wnt3a could still induce F-actin accumulation and changes in actin dynamics by targeting the remaining monomers of actin that have not been sequestered by Latrunculin B.

How is actin dynamics modulated during axon remodelling? Actin dynamics is important for proper axonal extension and axon guidance. Attractive molecules increase actin polymerisation and the levels of F-actin in growth cones within minutes, whereas repulsive guidance cues have the opposite effect [4,44–46]. During axon remodelling, however, the changes in the actin cytoskeleton are not well understood. Our time-lapse recordings of neurons expressing GFP-actin revealed that Wnt3a increased lamellar protrusion and filopodial velocity. Moreover, F-actin recovery experiments after depolymerisation with CytoD demonstrated that actin filaments polymerised faster in the presence of Wnt3a. Interestingly, short exposure to CytoD rapidly depolymerised accumulated F-actin in Wnt3a treated growth cones, but not in controls. Together these results indicate that Wnt3a induces the formation of highly dynamic actin filaments.

Wnts signal through a divergent Wnt canonical pathway to regulate the cytoskeleton during axon remodelling. Our previous studies have shown that Wnt3a through Dvl1 and inhibition of Gsk3**β,** but not transcription induces changes in the microtubule cytoskeleton during remodelling [14]. Similarly axon remodelling at the *Drosophila* NMJ has shown that Wg promotes microtubule reorganization at synaptic boutons through a pathway that requires Shaggy/Gsk3, but is transcription-independent [18]. Here we showed that Wnt3a signalling through Dvl1 and inhibition of Gsk3**β** was also involved in regulating the actin cytoskeleton. Thus, activation of a Wnt divergent canonical pathway is responsible for both actin and microtubule cytoskeletal changes during axon terminal remodelling.

Actin-binding proteins play a crucial role in the regulation of actin dynamics and organisation. Among the actin binding proteins is Eps8, a multi-functional protein that regulates the actin cytoskeleton through direct and indirect mechanisms [21,22,24,47]. Here we demonstrate that Eps8, which directly interacts with Dvl1 (this study and [36]), promotes growth cone enlargement, accumulation of F-actin and microtubule looping in growth cones, thus mimicking Wnt signalling in axon remodelling. Importantly, loss of function of Eps8 or interference of its interaction with Dvl1 blocks the ability of Wnt3a to induce axonal remodelling. How does Eps8 induce axonal remodelling? Previous studies showed that Eps8 acts as a capping protein to regulate filopodium and spine formation in hippocampal neurons [28–30]. Consistent with these findings, we found that Eps8 loss of function increased axonal filopodium density in DRG neurons. These results suggest that Eps8 could promote axonal remodelling through its actin capping activity. However, high capping activity would lead to a decrease in F-actin content [21,29,48] rather than an increase, as we observed in our Eps8 gain of function studies. These findings suggest that Eps8 induces axonal remodelling independently of its actin-capping activity. Eps8 has also been shown to confer actin bundling activity, resulting in excessive filopodium formation and membrane extension in heterologous cells [22,49]. In contrast, our gain of function experiments showed that Eps8 did not increase the number or the length of filopodia in growth cones (S4 Fig). Together these results suggest that Eps8 promotes terminal remodelling of axons through a mechanism independent of its capping or bundling activities.

How does Eps8 contribute to Wnt-mediated remodelling? Here we demonstrate that Dvl1, a molecular hub for Wnt signalling, directly interacts with Eps8. Importantly interfering with the interaction blocks Wnt-mediated remodelling. This interaction is through the PDZ domain, which is crucial for canonical Wnt-Gsk3β signalling [38–42], Interestingly, Eps8 has been shown to activate Akt, a kinase that phosphorylates and inactivates Gsk3β [50,51]. Indeed, Eps8 enhances cell proliferation and migration through the PI3K-Akt pathway and increases β-catenin levels [52–54]. Importantly, expression of a constitutively active form of Akt in DRG neurons increases branching and growth cone size [55], mimicking the effects observed upon activation of the Wnt3a-Dvl1-Gsk3β pathway [14]. These findings raise the possibility that Wnt3a regulates axonal remodelling through a pathway in which the interaction of Eps8 with Dvl1 activates Akt, leading to Gsk3β inhibition and resulting in growth cone enlargement and axon remodelling (Fig 7E).

In summary, our studies demonstrate that Wnt3a signalling, through the scaffold protein Dvl1 and inhibition of Gsk3β, promotes axon remodelling through changes in actin dynamics followed by changes in the microtubule cytoskeleton. Wnt3a-induced axonal remodelling is mediated by the actin-binding protein Eps8 through its direct interaction with Dvl1. Our studies reveal Eps8 as a novel target of Wnt signalling and demonstrate a role for Eps8 in axon terminal remodelling.

## Materials and Methods

### Cultured neurons

All experiments that included animals were carried out under personal and project licenses granted by the UK Home Office in accordance with the Animals (Scientific Procedures) Act 1986. All animal protocols were approved by the UK Home Office and the University College London Ethical Review Committee. Efforts were made to minimize animal suffering. DRG neurons were isolated from newborn mice or E18 rat embryos according to Kleitman *et al*. (1991) and plated at 180 cells/mm^2^ on coverslips coated with 1 mg/mL poly-L-lysine (Sigma) and 20 μg/mL Laminin (Sigma). Neurons were cultured in serum-free medium in the presence of 50 μg/mL NGF (Invitrogen). Two hours after plating neurons were treated with conditioned medium from control or Wnt3-HA expressing Rat1B cells or with 50 ng/mL of purified Wnt3a (R&D) or for two hours with 2μM Gsk3β cell-permeable peptide inhibitor (Calbiochem). For transfections, DRG neurons were electroporated using Amaxa nucleofection (Lonza) according to the manufacture’s instructions. Briefly, dissociated DRGs were centrifuged at 110g for 5 mins and re-suspended in the appropriate nucleofector solution containing 5–10 μg of DNA. Different DNA constructs were used: EGFP, GFP-β-actin, Dvl1-HA, Eps8-myc, scrambled shRNA and a cocktail of 3 shRNAs against Eps8 [29].

### Neuroblastoma 2a (N2a) cells

N2a cells (Sigma) were cultured in DMEM medium with Glutamax supplemented with 10% FBS and 1% v/v Pen/Strep. For transfections, cells were plated at 400 cells/mm^2^ and the following day were transfected using Lipofectamine 2000 (invitrogen), according to the manufacture’s instructions. Three hours after transfection, the medium was replaced with fresh Optimem medium and 2 hrs after 1 μM dibutyryl-cyclic-AMP (Sigma) was added to induce differentiation.

### Time-lapse recordings

DRG neurons were transfected with GFP-β-actin and treated with control or Wnt3a media two hours later. Time-lapse experiments were performed the following day on an inverted Axiovert Zeiss 200 microscope equipped with a heated stage and CO_2_ chamber. Recordings were three to five minutes long with a 20 seconds interval. Images were collected and analyzed using Metamorph software (Molecular Devices). Filopodia movement speed was determined by tracking their tips over consecutive frames, whereas the relative percentage of lamellar perimeter undergoing protrusion was quantified from time-lapse frames captured 1 min apart.

### Immunoprecipitation (IP)

N2a cells or spinal cord tissue were lysed in Triton buffer (50 mM Hepes, 100 mM NaCl, 4 mM EGTA, 2 mM MgCl_2_, 0.5% v/v TritonX100, pH 7.4) supplemented with 1 mM PMSF, 1 mM Na_3_VO_4_, 10 μg/mL leupeptin, 10 μg/mL aprotinin, 25 mM NaF and 1 μM pepstatin. Lysates were pre-cleared for 2 hours at 4°C (10 rpm) using G- or A- protein Sepharose beads. The pre-cleared lysates were then incubated overnight at 4°C (10 rpm) with specific anti-myc (Sigma) or anti-Dvl1 [13] antibodies or an anti-IgG cocktail (Biorad). The following day G- or A- protein Sepharose beads were added for 2 hours and subsequently centrifuged and washed 3 times with Triton buffer. Proteins bound on beads were extracted with equal volume of 2x SDS loading buffer (120 mM Tris, 100 mM DTT, 1.6 g/mL SDS, 0.4 g/mL bromophenol blue, 20% v/v glycerol, pH 6.8). Bead extracts were loaded on SDS/PAGE and antibodies against Dvl1 [13] (rabbit polyclonal, dilution 1:1000) or Eps8 (BD, mouse monoclonal, dilution 1:500) were used.

### Cloning

Dvl1 full-length cDNA was isolated by PCR and then cloned into the pGBDT7 vector using the EcoRI and SalI sites to create a GAL4 DNA binding domain (BD) Dvl1 fusion. Primers used were Fwd: 5’–CGTACAGAATTCGCGG AGACCAAAATCAT –3’ and Rvs: 5’–GGCTAGTCGACCATGATGTCCAC AAAG –3’. Eps8L3 full-length cDNA was isolated by PCR using cDNA made from P24 mouse brain and was then cloned onto the pGADT7 vector using the ClaI and XhoI sites to create a GAL4 activation domain (AD) fusion. Primers used were Fwd: 5’–AAGCTACTCGAGCTAATGAGTCATCCCCAGCATCCT –3’ and Rvs: 5’–AAGTACATCGATATGTCCCGGCCCAGCAGCA GAGCCAT –3’. Eps8-myc cloned into the PCS2+ was a kind gift from Dr J. Miller (University of Minnesota, Minneapolis). To create the Eps8Δ533–821 the Eps8-myc was used as a template for a PCR reaction, Fwd: 5’–AAGTACATCGATATGAATGG TCATATGTCTAACCGC– 3’ and Rvs: 5’–AAGTACCTCGAGTCATAGGT CTTCGGAGATTAGCTTTTGCTCG– 3’. The PCR product was inserted into PCS2+ using the ClaI and XhoI sites. The cloning of the Dvl1ΔPDZ has been previously described in [56]. To clone the PDZ domain of Dvl1 (amino acids 278–337) the mouse Dvl1 cDNA was used as a template for a PCR reaction, Fwd: 5’–CGTACAGAATTCATGATCTACATTGGATCCATCAT– 3’ and Rvs: 5’–GCTATACTCGAGTCAAGCGTAATCTGGAACATCGTACTTGGCCTCTGTGA GACTGAT– 3’. The PCR product was inserted into PCS2+ using the EcoRI and XhoI sites. All constructs were verified by DNA sequencing.

### Yeast two hybrid screen and assays

To perform the yeast two hybrid screen the AH109 yeast strain (MATa), transformed with the GAL4 DNA binding domain (BD) Dvl1 fusion, was mated with the Y187 strain (MATα), transformed with a cDNA library isolated from adult mouse brain (Clonetech), according to the manufacture’s instructions. For the yeast two hybrid assays empty pGBKT7 and GAL4 DNA binding domain (BD) Dvl1 fusion were transformed into the yeast strain AH109 (MATa), whereas the empty pGADT7 and Eps8L3-GAL4 activation domain (AD) fusion were transformed into the Y187 strain (MATα). Two yeast transformants for each plasmid combination were mated on rich YPDA medium and selected under nutrition restriction on plates containing synthetic media without Leucine and Tryptophan (Clontech). Protein interactions were then assayed by monitoring growth on synthetic media lacking Histidine or Histidine and Adenine (Clontech).

### Immunofluorescence microscopy and analyses

Cultures were fixed with 4% PFA / 4% sucrose in PBS for 20 mins at room temperature. Primary antibodies against HA (Roche, rat monoclonal, dilution 1:1000), GFP (Upstate, chicken polyclonal, dilution 1:500), Myc (Sigma, rabbit polyclonal, dilution 1:2000) were used. Secondary antibodies conjugated with Alexa 488, Alexa 568, Alexa 647 and Phalloidin conjugated with Alexa 647 were from Molecular Probes. Fluorescent images of growth cones from NT3-rensponsive neurons were captured using an Olympus BX60 wide-field microscope with a 100x oil objective (NA = 1.30). At least 50 growth cones were analysed per condition using the Metamorph software (Molecular Devices). Growth cone size was determined from the growth cone area, measured manually using the drawing tool. Accumulation of F-actin was determined by setting a high threshold on the Phalloidin images (red and white area in pseudocolor images).

### Statistical analyses

Values given are mean ± standard error. Values given are mean ± error. Data presented is the pool from at least three independent experiments. For datasets with normal distribution, an ANOVA test was used. For datasets that were not normally distributed, the Kruskal-Wallis test was used.

## Supporting Information

S1 Fig Dvl1 overexpression in DRG growth cones. HA expression in growth cones presented in Fig 4A. Scale bar: 5 μm.
(EPS)

S2 Fig Eps8 gain of function induces the formation of looped microtubules in growth cones. (A) Myc expression in growth cones presented in Fig 6A. (B) Eps8 expressing DRG neurons possess enlarged growth cones with looped microtubules.
(EPS)

S3 Fig Loss of function of Eps8 induces filopodium formation in axons. (A and B) Expression of Eps8 shRNAs significantly reduces the levels of endogenous Eps8 in growth cones. Scale bar: 5 μm. (C and D) Eps8 KD significantly increases the number of axonal filopodia. Scale bar: 2 μm. **p<0.01.
(EPS)

S4 Fig Eps8 does not promote filopodium formation in growth cones. (A and B) Eps8 gain of function does not increase the length or the number of filopodia in growth cones. *p<0.05.
(EPS)

S5 Fig Original blots from IP experiments. (A) Blot presented in Fig 5C. (B) Blot presented in Fig 5D. (C) Blots from Fig 7B. Molecular size for ladder bands (From top to bottom): 175kDa, 80kDa, 58kDa, 46kDa, 30kDa and 23kDa.
(EPS)

S1 Movie Time-lapse recording of a growth cone expressing GFP-actin treated with control growth medium. A GFP-actin expressing growth cone from control-treated DRG neurons was recorded for 3 mins with a frame interval of 20 seconds. The perimeter of lamella undergoing protrusion and filopodium velocity was quantified (% lamellar protrusion: 19.58 ± 2.99; filopodium velocity: 0.031 ± 0.001 μm/sec).
(MOV)

S2 Movie Time-lapse recording of a growth cone expressing GFP-actin treated with Wnt3a growth medium. A GFP-actin expressing growth cone from Wnt3a-treated DRG neurons was recorded for 3 mins with a frame interval of 20 seconds. The perimeter of lamella undergoing protrusion and filopodium velocity was quantified (% lamellar protrusion: 32.77 ± 1.15; filopodium velocity: 0.057 ± 0.008 μm/sec).
(MOV)
